# Association between Vaccination Status for COVID-19 and the Risk of Severe Symptoms during the Endemic Phase of the Disease

**DOI:** 10.3390/vaccines11101512

**Published:** 2023-09-22

**Authors:** Oliver Mendoza-Cano, Xóchitl Trujillo, Mónica Ríos-Silva, Agustin Lugo-Radillo, Verónica Benites-Godínez, Jaime Alberto Bricio-Barrios, Herguin Benjamin Cuevas-Arellano, Eder Fernando Ríos-Bracamontes, Walter Serrano-Moreno, Yolitzy Cárdenas, Efrén Murillo-Zamora

**Affiliations:** 1 Facultad de Ingeniería Civil, Universidad de Colima, km. 9 Carretera Colima-Coquimatlán, Coquimatlán 28400, Mexico; 2 Centro de Estudios e Investigación en Biocultura, Agroecología, Ambiente y Salud Colima, Ex-Hacienda Nogueras S/N, Nogueras 28450, Mexico; 3 Centro Universitario de Investigaciones Biomédicas, Universidad de Colima, Av. 25 de Julio 965, Colima 28045, Mexico; 4 CONAHCyT—Centro Universitario de Investigaciones Biomédicas, Universidad de Colima, Av. 25 de Julio 965, Colima 28045, Mexico; 5 CONAHCyT—Faculty of Medicine and Surgery, Universidad Autónoma Benito Juárez de Oaxaca, Ex Hacienda Aguilera S/N, Carr. a San Felipe del Agua, Oaxaca 68020, Mexico; 6 Coordinación de Educación en Salud, Instituto Mexicano del Seguro Social, Calzada del Ejercito Nacional 14, Tepic 63169, Mexico; 7 Unidad Académica de Medicina, Universidad Autónoma de Nayarit, Ciudad de la Cultura Amado Nervo, Tepic 63155, Mexico; 8 Facultad de Medicina, Universidad de Colima, Av. Universidad 333, Colima 28040, Mexico; 9 Facultad de Ciencias, Universidad de Colima, Bernal Díaz del Castillo 340, Colima 28045, Mexico; 10 Departamento de Medicina Interna, Hospital General de Zona No. 1, Instituto Mexicano del Seguro Social, Av. Lapislázuli 250, Villa de Álvarez 28984, Mexico; 11 Unidad de Investigación en Epidemiología Clínica, Instituto Mexicano del Seguro Social, Av. Lapislázuli 250, Villa de Álvarez 28984, Mexico

**Keywords:** COVID-19, vaccines, cohort studies, polymerase chain reaction, Mexico

## Abstract

The global health emergency caused by COVID-19 concluded in May 2023, marking the beginning of an endemic phase. This study aimed to evaluate the association between vaccination status and other patient characteristics and the risk of severe disease during this new endemic period. A nationwide cohort study was conducted in Mexico, where we analyzed data from 646 adults who had received positive confirmation of COVID-19 through PCR testing from May to August 2023. The overall risk of severe symptoms in the study sample was 5.3%. The average time elapsed from the last vaccine shot to symptom onset was over six months in all the immunized groups (1, 2 or 3 vaccine doses). Compared to unvaccinated patients, those with three vaccine doses showed an elevated risk of severe symptoms. Advancing age and various chronic comorbidities (specifically cardiovascular, kidney, and obstructive pulmonary conditions) were associated with a heightened risk of severe COVID-19 manifestations. These findings underscore the ongoing seriousness of COVID-19, even in an endemic phase, underscoring the urgent need for tailored interventions aimed at high-risk patients.

## 1. Introduction

The emergence of the COVID-19 pandemic, resulting from the infection with severe acute respiratory syndrome coronavirus 2 (SARS-CoV-2), had high social and economic implications [1]. Concurrently with vaccine development, significant progress was achieved in reducing virus transmission and mitigating its virulence [2]. Consequently, the global health emergency classification associated with COVID-19 was lifted, signifying the shift of the disease into an endemic stage [3].

Mexico’s COVID-19 vaccination plan was characterized by a tiered approach prioritising high-risk populations and essential workers [4]. The initial phases, which started on 24 December 2020 [5], focused on healthcare personnel, the elderly population, and individuals with underlying health conditions, acknowledging their increased vulnerability to severe COVID-19 outcomes [6]. As vaccine availability allowed, the strategy expanded to encompass broader demographic groups, ultimately targeting a substantial proportion of the population. By the end of the first quarter of 2023, nearly 78% of the country’s total population had received at least one dose of a COVID-19 vaccine [7].

In Mexico, a total of eight COVID-19 vaccines were authorized and administered. These vaccines include BNT162b2 (Pfizer-BioNTech; New York, NY, USA–Mainz, Germany), AZD1222 (AstraZeneca; Cambridge, UK), Ad26.COV2-S (Janssen-CILAG; Beerse, Belgium–Schaffhausen, Switzerland), CX-024414 (Moderna; Cambridge, MA, USA), BBIBP-CorV (Sinopharm; Shanghai, China), CoronaVac (Sinovac Biotech; Beijing, China), BBV152 (Bharat Biotech; Hyderabad, India), and Ad5-nCoV (CanSino Biologics, Tianjin, China) [8].

The post-global emergency phase resulting from COVID-19 is marked by the prevailing presence of the XBB.1.5 subvariant of Omicron SARS-CoV-2, recognized for its heightened transmissibility [9]. Evaluating predictors of severe COVID-19 during the endemic phase of the disease, including the effect of vaccination status, holds critical importance for effective public health planning and resource allocation [10].

In addition, the identification of predictors can aid in refining risk stratification models, enabling healthcare systems to allocate medical interventions better and prioritize vulnerable individuals [11]. Finally, insights into predictors of severe outcomes can guide the development of targeted preventive strategies and therapeutic interventions, potentially reducing the burden on healthcare infrastructure and minimizing the impact of the disease on individuals and communities [12]. However, and to the best of our knowledge, no published studies have comprehensively evaluated predictors of severe symptoms after the conclusion of the COVID-19 emergence.

Hence, this study aimed to assess the effect of COVID-19 vaccination status on the risk of severe laboratory-confirmed illness in adults during the endemic phase of the disease. Furthermore, the study evaluated the association between different patient characteristics and the risk of severe manifestations. Our hypothesis posited that vaccinated adults, compared to their unvaccinated counterparts, might experience a decreased risk of severe symptoms during the endemic phase of COVID-19.

## 2. Materials and Methods

We conducted a nationwide and retrospective cohort study in Mexico during August 2023. Potentially eligible subjects (comprising laboratory-confirmed COVID-19 cases using reverse-transcription polymerase chain reaction, RT-PCR) were sourced from nominal records within an epidemiological surveillance system for viral respiratory diseases, targeting SARS-CoV-2, influenza virus, and other pathogens of public health concern. This system, known as SINOLAVE, primarily sourced data from patients’ medical records and, when applicable, death certificates. SINOLAVE is managed by the Mexican Institute of Social Security (IMSS), which provides healthcare services to approximately 60% (72 million people) of Mexico’s total population [13].

Adult patients aged 18 years and above, who exhibited respiratory symptoms suggestive of COVID-19 between 5 May and 10 August 2023, were eligible. Patients with a negative RT-PCR test result and those lacking complete clinical and epidemiological data of interest were excluded. A total of 172 patients under 18 years of age and 21 patients with insufficient data of interest were excluded.

In accordance with normative guidelines [14], clinical specimens obtained as nasopharyngeal or deep nasal swabs were employed for RT-PCR testing. Nucleic acids were extracted from a 200 μL sample using the MagNa Pure LC Total Nucleic Acid Isolation Kit automated system (catalog: 03038505001; Roche Diagnostics, Mannheim, Germany), following the protocols detailed in the study by Fernandes-Matano et al. [15]. Detection of SARS-CoV-2 was performed using the primers and probes proposed by Corman et al. [16], utilizing the SuperScript III Platinum One-step qRT-PCR System (catalog: 12574035; Invitrogen Carlsbad, CA, USA) in conjunction with the 7500 Fast Real-Time PCR System (Applied Biosystems, Foster City, CA, USA). The analytical procedure was carried out within the network of laboratories dedicated to epidemiological surveillance under the jurisdiction of the IMSS.

Demographic and clinical variables were extracted from the audited surveillance system. Vaccination status was assessed based on the number of COVID-19 vaccine doses received (none, 1, 2, or 3). When applicable, the dates of vaccine shots were utilized to calculate the number of days elapsed between the last shot and the onset of symptoms. The analyzed comorbidities were determined by physician-diagnosed history (no/yes). The following conditions were assessed: current tobacco use, obesity (defined by a body mass index [BMI] of 30 or above), asthma, chronic obstructive pulmonary disease (COPD), type 2 diabetes mellitus, cardiovascular disease, chronic kidney disease (CKD), and immunosuppression (due to any cause).

Severe COVID-19, our primary binary outcome (no/yes), was defined by the occurrence of pneumonia. The latter was characterized by the presence of respiratory clinical symptoms (including cough, fever, dyspnea, and chest pain), coupled with radiographic evidence of pneumonia (evidenced by bilateral ground glass opacities or consolidations visible on computed tomography [CT] or X-ray) necessitating hospitalization [14].

Summary statistics were calculated. We utilized risk ratios (RR) and 95% confidence intervals to evaluate the association between vaccination status and other exposures of interest with the risk of severe COVID-19 manifestations. These estimations were derived using generalized linear regression models. We constructed 11 models, one for each independent variable and a final multiple model that incorporated all explanatory variables. In the construction of the multiple regression model, we adhered to the Hosmer and Lemeshow approach. Thus, variables that exhibited a p-value of <0.2 in bivariate analysis, or those identified as potential confounders based on scientific rationale, were included, irrespective of their p-value [17].

In the generalized linear regression analysis context, the unvaccinated group was designated as the reference category for calculating the relevant estimates. This choice of reference category was made to establish a baseline for comparing vaccination effects [18], as previously employed during the COVID-19 emergency [19,20,21]. Such an approach aligns with the fundamental principles of causality in epidemiology [22] and ultimately facilitates a more robust assessment of the vaccine’s efficacy in reducing the risk of severe outcomes [23].

This study obtained approval from the Local Committee of Ethics in Health Research (601) of the IMSS (approval R-2023-601-015). None of the participants were physically present or interviewed during any phase of this study, and all researchers adhered to stringent ethical guidelines.

## 3. Results

**Figure 1:**
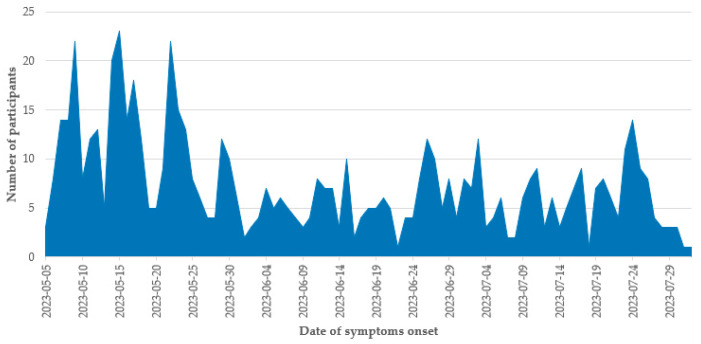
Date of symptoms onset of enrolled subjects, Mexico 2023.

Data from 646 patients with laboratory-confirmed COVID-19 were analyzed. As presented in Figure 1, approximately half (47.2%, n = 305/646) of the included adults experienced symptom onset in May 2023. Nearly two-thirds (62.5%, n = 404/646) of the participants were female, and the overall median age was 44 years and ranged (interquartile range) from 29 to 60 years old. The overall risk of severe symptoms in the study sample was 5.3% (n = 34/646).

The average time interval between the date of the final vaccine administration and the onset of symptoms was 304.3 ± 111.4 days for individuals who had received one dose, 218.7 ± 63.3 days for those who had received two doses, and 202.1 ± 49.7 days for individuals who had received a vaccine booster (third dose). Participants who experienced severe COVID-19, when compared to those with non-severe disease, were found to have a higher likelihood of being vaccinated (at least one dose: 38.2% vs. 17.2%, p = 0.002). This difference was particularly more pronounced among those who had received three vaccine doses (23.5% vs. 3.9%, p < 0.001). 

As presented in Table 1, patients with severe symptoms had a higher likelihood of being male and were older than those with non-severe COVID-19. They also had a higher prevalence of a personal history of the assessed chronic comorbidities, specifically COPD (17.7% vs. 1.8%, p < 0.001), type 2 diabetes mellitus (38.2% vs. 13.2%, p < 0.001), cardiovascular disease (14.7% vs. 1.5%, p < 0.001), CKD (14.7% vs. 2.8%, p < 0.001), and immunosuppression (8.8% vs. 1.5%, p = 0.002). 

In the multiple generalized linear regression model presented in Table 2, compared to unvaccinated participants, those who had received three vaccine doses revealed a 19% increased risk of severe disease (RR = 1.19, 95% CI 1.11–1.28, p < 0.001). The estimates for the remaining categories, involving one or two vaccine shots, did not demonstrate statistical significance.

Patients with COPD exhibited the highest risk of severe COVID-19 (RR = 1.23, 95% CI 1.11–1.37, p = 0.004), followed by the risk associated with a personal history of cardiovascular disease (RR = 1.19, 95% CI 1.06–1.33, p = 0.004), and CKD (RR = 1.12, 95% CI 1.02–1.23, p = 0.020). The study sample did not yield any other significant associations.

## 4. Discussion

Our findings suggest that SARS-CoV-2 vaccination may not provide enduring protection against severe disease, preventing us from confirming our initial hypothesis. Furthermore, we observed that patients who received three vaccine doses were more likely to develop severe symptoms. However, as the remaining vaccinated groups (1 or 2 vaccine shots) did not show an elevated risk for severe disease compared to unvaccinated adults, this finding should be interpreted cautiously. To gain a more comprehensive understanding of the vaccine’s effects in the endemic phase of COVID-19, further research with a larger sample size is imperative.

The presented results underscore that specific patient-related factors, especially age and the presence of pre-existing chronic conditions, significantly contribute to an elevated risk of encountering severe cases of COVID-19 during the endemic phase of the illness. The association between advancing age and the presence of chronic diseases with an escalated risk of severe disease has been widely documented throughout the entire course of the pandemic’s progression [24,25].

These insights highlight the need for tailored and focused public health strategies that prioritize managing and caring for individuals burdened with chronic health conditions [26]. Such an approach is pivotal for alleviating the impact of severe cases and optimizing patient outcomes within the evolving framework of the endemic phase.

Within our study sample, individuals who had received a vaccine booster were at increased risk for severe disease compared to those who remained unvaccinated. It is worth noting, however, that the mean interval between the vaccination date and the onset of symptoms extended beyond six months for participants who had received a booster shot and those who had been administered one or two vaccine doses. Consequently, it was anticipated that the antibody titers would fall below protective levels [27].

Additionally, it is important to highlight that the median age of patients who had received three vaccine doses (48, interquartile range [IQR] 37–66 years old) was higher (*p* = 0.047) than that of unvaccinated individuals (44, IQR 29–60 years old) (data not presented). These factors may collectively contribute to the observed scenario, and the documented association must be interpreted cautiously.

As of March 2023, about three-quarters (77.5%) of the Mexican population had received at least one dose of the COVID-19 vaccine [7]. However, in our study sample, the vaccine coverage (at least one dose) was 18.2%. Taking into account Mexico’s high disease burden during the critical phases of the pandemic emergency [28], the fact that our study exclusively focused on enrolling symptomatic cases, and considering the prolonged effectiveness of active immunization in comparison to passive immunization [29,30,31], we hypothesize that previous SARS-CoV-2 infections might exert an undisclosed influence on the observed vaccination rates within our study. 

This latter hypothesis is based on the concept that individuals who have recuperated from previous infections might perceive a unique risk-benefit equilibrium in relation to vaccination [32,33]. Such a perception could potentially underlie the observed fluctuations in uptake rates, as outlined in our analysis. These factors underscore the complex interplay among natural immunity, vaccination approaches, and public attitudes. Consequently, further investigation is warranted to elucidate its potential influence on vaccination campaigns and the broader population’s immunity landscape.

Our study did not observe a significant association between obesity, defined by a BMI of 30 or higher, and the risk of severe symptoms [34]. We hypothesize that this lack of association may be attributed to an unexpectedly low prevalence of obesity among individuals with mild symptoms (11.3%) and severe disease (20.6%). According to the latest (2022) National Health and Nutrition Survey, the overall prevalence of obesity among Mexican adults stands at 36.9% [35]. Further research is needed to understand the relationship between obesity and severe COVID-19 during this endemic phase.

A personal history of asthma was associated with the risk of severe symptoms in the bivariate analysis but did not maintain significance in the multiple models. This observation aligns with findings reported in prior published studies [36,37,38].

The incorporation of cases verified through RT-PCR testing is a major strength of this study. Nevertheless, it is essential to acknowledge and address potential limitations inherent in our research methodology. First, our research did not encompass an evaluation of the antibody titers within vaccinated adults. Consequently, we could not evaluate a correlation between these antibody levels and the risk of experiencing severe manifestations of COVID-19. This aspect represents a gap in our study that warrants consideration in future research endeavors.

Second, the personal history of COPD was associated with a 23% escalation in the risk of severe COVID-19 symptoms (RR = 1.23, 95% CI 1.11–1.37; p < 0.001). This observed increase in risk constitutes the highest effect within our study cohort. However, the exposure assessment was conducted as a dichotomous variable, thereby excluding the inclusion of other pertinent clinical data, such as disease staging. The absence of these comprehensive factors may limit the depth of our conclusions and warrant further exploration.

Third, it is pertinent to recognize the contextual influence of the pandemic’s evolution. The elevated likelihood of previous SARS-CoV-2 infections due to the pandemic’s progression potentially introduces a confounding factor that might have impacted our obtained results. Pre-existing anti-SARS-CoV-2 nucleocapsid antibodies could have been detected through a straightforward serological test, indicating a previous SARS-CoV-2 infection. The interplay between prior infections and vaccination status might be complex and could contribute to variations in the severity of outcomes.

Fourth, we lacked genomic data pertaining to the specific SARS-CoV-2 variant and subvariant implicated in each analyzed individual. Nevertheless, relying on authoritative information sourced from the genomic surveillance of COVID-19 in Mexico, it is apparent that the XBB.1.5 (‘Kraken’) subvariant of the Omicron lineage was notably prevalent throughout the study period [39].

Fifth, information regarding concurrent infections (i.e., bacterial or viral) is not systematically collected by the analyzed system, so we cannot assess its impact on the risk of severe manifestations in the analyzed cohort.

## 5. Conclusions

As the world transitions into an era wherein COVID-19 evolves into a persistent public health challenge, prioritizing tailored approaches aimed at safeguarding and aiding these susceptible populations should continue to stand as a paramount objective for healthcare systems and policymakers. Our study may enrich our comprehension of the evolving dynamics characterizing COVID-19 within an endemic phase. The heightened susceptibility to severe manifestations among specific groups accentuates the significance of upholding vigilant surveillance protocols, bolstering vaccination initiatives, and devising healthcare strategies that effectively attend to the distinct requirements of individuals at an elevated risk.

## Figures and Tables

**Table 1:** Characteristics of the study sample for selected variables, Mexico 2023.

Characteristic	Severe COVID-19		p
	No	Yes	
Gender			
Female	390 (63.7)	14 (41.2)	0.008
Male	222 (36.3)	20 (58.8)	
Age (years, median and IQR)	43 (28–58)	68 (59–76)	<0.001
COVID-19 vaccination status			
Not vaccinated	507 (82.8)	21 (61.8)	<0.001
One dose	19 (3.1)	0 (0)	
Two doses	62 (10.1)	5 (14.7)	
Three doses	24 (3.9)	8 (23.5)	
Personal history of:			
Smoking (current), yes	21 (3.4)	4 (11.8)	0.014
Obesity (BMI ≥ 30), yes	69 (11.3)	7 (20.6)	0.101
Asthma, yes	10 (1.6)	1 (2.9)	0.566
COPD, yes	11 (1.8)	6 (17.7)	<0.001
Type 2 diabetes mellitus, yes	81 (13.2)	13 (38.2)	<0.001
Cardiovascular disease, yes	9 (1.5)	5 (14.7)	<0.001
CKD, yes	17 (2.8)	5 (14.7)	<0.001
Immunosuppression (any cause), yes	9 (1.5)	3 (8.8)	0.002

Abbreviations: COVID-19, coronavirus disease 2019; IQR, interquartilic range; BMI, body mass index; COPD, chronic pulmonary obstructive disease; CKD, chronic kidney disease. Notes: (1) The absolute frequencies (n) and relative frequencies (%) are presented unless specified as the median; (2) The *p*-values from chi-squared or U-test are reported accordingly.

**Table 2:** Factors associated with the risk of severe COVID-19, Mexico 2023.

Characteristic	RR (95% CI), p	
	Bivariate Analysis	Multivariate Analysis
Gender		
Female	1.00	1.00
Male	1.05 (1.01–1.09), 0.008	1.03 (0.99–1.07), 0.082
Age (per each additional year)	1.003 (1.001–1.004), <0.001	1.002 (1.001–1.003), <0.001
COVID-19 vaccination status		
Not vaccinated	1.00	1.00
One dose	0.96 (0.87–1.06), 0.437	0.95 (0.87–1.05), 0.342
Two doses	1.04 (0.98–1.09), 0.220	1.02 (0.97–1.08), 0.345
Three doses	1.23 (1.14–1.33), <0.001	1.19 (1.11–1.28), <0.001
Personal history of:		
Obesity (BMI ≥ 30)		
No	1.00	1.00
Yes	1.05 (0.99–1.10), 0.101	1.04 (0.99–1.09), 0.135
Smoking (current)		
No	1.00	1.00
Yes	1.12 (1.02–1.22), 0.014	1.08 (0.99–1.17), 0.076
Type 2 diabetes mellitus		
No	1.00	1.00
Yes	1.11 (1.05–1.16), <0.001	1.02 (0.97–1.07), 0.404
Cardiovascular disease		
No	1.00	1.00
Yes	1.37 (1.22–1.53), <0.001	1.19 (1.06–1.33), 0.004
COPD		
No	1.00	1.00
Yes	1.36 (1.23–1.51), <0.001	1.23 (1.11–1.37), <0.001
Asthma		
No	1.00	1.00
Yes	1.02 (1.01–1.03), <0.001	1.04 (0.92–1.18), 0.556
CKD		
No	1.00	1.00
Yes	1.20 (1.09–1.32), <0.001	1.12 (1.02–1.23), 0.020
Immunosuppression (any cause)		
No	1.00	1.00
Yes	1.22 (1.08–1.39), 0.002	1.09 (0.96–1.23), 0.185

Abbreviations: COVID-19, coronavirus disease 2019; RR, risk ratio; CI, confidence interval; BMI, body mass index; COPD, chronic pulmonary obstructive disease; chronic kidney disease. Notes: (1) Generalized linear regression models were used to obtain the reported estimates; (2) The estimates from the multiple regression model were adjusted for all the variables presented in the table; (3) In the context of COVID-19 vaccination status, the unvaccinated group was selected as the reference to quantify the change in risk associated with receiving the vaccine.

## Data Availability

The data and materials analyzed in this study are available from the corresponding author upon request.
